# On the Use of Mercury in the Treatment of Southern Fevers

**Published:** 1845-05

**Authors:** Lafayette Carr

**Affiliations:** Aberdeen, Mississippi; Louisville, Ky.


ART. II.—*On the Use of Mercury in the Treatment of Southern Fevers.* By LAFAYETTE CARR, M. D., of Aberdeen, Mississippi.*

There is perhaps no subject connected with practical medicine upon which the sentiments of physicians are undergoing a more rational change than the exhibition of mercury in our Southern fevers. If the fact were not so I should probably incur the charge of presumption in expressing some of the opinions contained in this essay. I do not mean, however, to exclaim against the judicious employment of the remedy, for none can be more fully impressed with a sense of its utility when used with proper discretion, than I am. But my design is simply to point out the circumstances which, as I conceive, render its use improper, and likewise to show in what manner it has been abused. It cannot be denied that to meet certain indications in the treatment of fever, mercury is an admirable remedy. This is a fact which the experience of every day in practice confirms; while it is equally plain that its exhibition under other circumstances is preposterous and calculated to do mischief. In those cases of fever that seem to depend on torpor of the secretory organs alone, nothing can be more admirable than the action of calomel or blue mass in exciting them to a healthy exercise

---

* The author of the following Essay has been engaged for several years in the practice of physic in Mississippi, and his experience in the use of mercury in the fevers of that region was embodied in an inaugural dissertation submitted to the Managers and Faculty of the Medical Institute of Louisville for the degree of doctor of medicine.—EDS.

of their functions, and whenever we find suppressed secretion unconnected with excessive excitement, we may confidently expect beneficial effects from its operation. But such is not very often the case in our climate, for here we scarcely see a case in which suppressed secretion is unconnected with excessive excitement either local or general, and either the one or the other of these circumstances, I apprehend, may constitute an objection to its employment. For it has appeared to me that much of the inordinate action of the medicine is produced by the inflammatory nature of our fevers; consequently the propriety of using mercury in their treatment previous to depletion, is at least questionable. It is customary to prescribe mercury as well in excessive excitement of the secretory organs as in torpor of those organs, but this practice is of exceedingly doubtful propriety. Indeed, it seems to me an absurdity to employ the same agent to meet such opposite indications in practice. True, by its secondary operation as an evacuant, it will sometimes answer our expectations, but these are cases in which the excess of local action is not very great, or is unaccompanied by that disposition to inflammatory action in the system at large, which usually marks our fevers. They are therefore to be regarded as an exception to the general rule, and in most of these cases perhaps a sufficient impression could be made on the secretions and our purpose fully answered by the exhibition of some of the milder cathartics. For we should not limit our views of the operation of mercury to its action upon the secretions alone, but bear in mind that it is a stimulant which affects every fibre of the system; and one thing has impressed me with the force of truth, that when mercury is given in the commencement of our fevers without previous depletion as a preliminary step, it will generally do harm. It is too common to prescribe mercury to restore the secretions without a due consideration of the circumstances attending their suppression; and whenever this course is pursued in the commencement of our fevers, it will most commonly be attended with disagreeable results. So far from restoring the secretions when given in cases marked by excessive excitement, it commonly deranges them still more materially by increasing the general excitement, and often by the development of excessive local irritation in the organs upon which it specifically acts. These diseases appear to throw the system into that state of morbid irritation that is peculiarly favorable to the development of inflammatory action, which action, mercury by its universally stimulating effects rarely fails to produce, and being generally accompanied with a preponderance of morbid action in one particular part, the whole irritating effects of the remedy are diverted to that part, and thus the disposition of the disease to locate itself is confirmed. We often see fevers wherein the liver appears to be already in a state of great irritation with a superabundant secretion of bile, and in such fevers nothing is more common than to prescribe calomel forthwith, and without a more attentive examination of the case. At our next visit, perhaps, we shall find the system thrown into the most violent commotion; the skin and conjunctiva become icterode, copious discharges of vitiated bile take place by stool, and yet the fever and sufferings of the patient are greatly increased. If the use of the medicine is still persisted in with a view of exciting ptyalism, high inflammatory action is developed in the salivary glands, tongue, mouth, &c., which not unfrequently ends in mortification and death. In such cases venesection ought certainly to precede the use of mercury, but the propriety of its exhibition may be questioned even after this precaution, for if we bleed in the first place to remove irritation, either local or general, why should we the next moment prescribe an agent that will induce it again?

Nor is its administration in our congestive fevers exempt from the same necessary precautions. Here excitement is so intimately and mysteriously connected with debility that the operation of every remedy is perhaps attended with uncertainty. Much of the mischief which results from the employment of this agent is no doubt owing to culpable negligence in permitting it to remain in the system until it has been absorbed. Now congestive fever as it occurs with us is a disease in which purgatives must be used with extreme caution, and if the practitioner has unwarily given mercury to a considerable extent with a view of restoring the secretions by its action, he not only fails to accomplish this object but often finds himself in an embarrassing dilemma, to clear the system of the medicine before absorption has taken place, and at the same time observe a proper degree of caution in his measures for the safety of his patient. Sometimes it is true when the stomach and bowels are not particularly implicated, the remedy will restore the secretions and relieve him from embarrassment, but such instances are of unfrequent occurrence, and where those organs are chiefly concerned calomel, by its local irritation, will only increase the disease.

But it is in fevers characterized by high mucous irritation throughout their course, that the injudicious employment of mercury is most to be regretted, because here its effects are masked by the insidious type of the disease. The injurious effects of mercury upon the stomach and bowels when given in considerable quantities even where no previous disposition to disease existed in these organs is a well known fact. From this we may infer that the practice of giving it in alterative doses throughout the course of a disease marked by a subacute inflammation of these parts, is preposterous. That it is so, a careful observation of cases treated in this way will convince us. The first operation of the medicine seems to be beneficial, and this is the more unfortunate as the physician deceived by the partial cleansing of the tongue and abatement of the fever, thinks that a continuation of the medicine alone is necessary to effect his purpose. But soon the tongue becomes still more dry and red, the fever rises a grade higher, and the patient grows more irritable, and here the physician is apt to commit another mistake by presuming all this irritation to be caused by the inveteracy of the complaint, and discovering neither soreness of the mouth nor fœtor of the breath, he continues to exhibit mercury until the appearance of an eruption on the skin and mucous membrane discloses his error, or the patient sinks exhausted by the insidious irritation of an agent that was thought to afford him the greatest chance for recovery. In such cases it seems to create a morbid irritation in the system, at the same time that it irritates and perverts the tone of the stomach and bowels.

Formerly it was thought that to secure the safety of the patient it was only necessary to induce salivation, and accordingly calomel was given in the most extraordinary quantities and frequently with the most deplorable results. This liberal and profuse use of a violent mineral poison having received the sanction of physicians became the common practice not only of physicians themselves, but unfortunately also of others entirely ignorant of its therapeutic agency, and was followed, as a matter of course, generally by serious and sometimes by fatal consequences. Hence the popular prejudice against it at this time, and hence that predilection for quackery and empiricism which seems to be characteristic of the age. We now understand that the substitution of one disease will not always remove, but will sometimes aggravate an existing one, and thus we may account for the failure of mercury to cure in many instances that seem to indicate its use.

The abuse of a remedy can form no valid objection to its judicious use, but it is evident that there has been and still continues to be an abuse of this remedy which the interests of science as well as of humanity require should be corrected.

Louisville, Ky., March, 1845.

